# Epigenome mapping highlights chromatin-mediated gene regulation in the protozoan parasite *Trichomonas vaginalis*

**DOI:** 10.1038/srep45365

**Published:** 2017-03-27

**Authors:** Min-Ji Song, Mikyoung Kim, Yeeun Choi, Myung-hee Yi, Juri Kim, Soon-Jung Park, Tai-Soon Yong, Hyoung-Pyo Kim

**Affiliations:** 1 Department of Environmental Medical Biology, Institute of Tropical Medicine, Yonsei University College of Medicine, Seoul, 03722, Korea; 2 Graduate Program of Nano Science and Technology, Yonsei University College of Medicine, Seoul, 03722, Korea; 3 BK21 PLUS Project for Medical Science, Yonsei University College of Medicine, Seoul, 03722, Korea

## Abstract

*Trichomonas vaginalis* is an extracellular flagellated protozoan parasite that causes trichomoniasis, one of the most common non-viral sexually transmitted diseases. To survive and to maintain infection, *T. vaginalis* adapts to a hostile host environment by regulating gene expression. However, the mechanisms of transcriptional regulation are poorly understood for this parasite. Histone modification has a marked effect on chromatin structure and directs the recruitment of transcriptional machinery, thereby regulating essential cellular processes. In this study, we aimed to outline modes of chromatin-mediated gene regulation in *T. vaginalis*. Inhibition of histone deacetylase (HDAC) alters global transcriptional responses and induces hyperacetylation of histones and hypermethylation of H3K4. Analysis of the genome of *T. vaginalis* revealed that a number of enzymes regulate histone modification, suggesting that epigenetic mechanisms are important to controlling gene expression in this organism. Additionally, we describe the genome-wide localization of two histone H3 modifications (H3K4me3 and H3K27Ac), which we found to be positively associated with active gene expression in both steady and dynamic transcriptional states. These results provide the first direct evidence that histone modifications play an essential role in transcriptional regulation of *T. vaginalis*, and may help guide future epigenetic research into therapeutic intervention strategies against this parasite.

*Trichomonas vaginalis* is a microaerophilic, single-cell flagellate of the phylum Parabasalia, one of the earliest diverging eukaryotic lineages^1^. Comprising only a single stage, the life cycle of *T. vaginalis* is quite simple: a trophozoite that reproduces by simple binary fission^2^. This organism resides in the urogenital tracts of both men and women and causes trichomoniasis, the most common non-viral sexually transmitted disease worldwide^2^,^3^. Trichomoniasis leads to vaginitis in women and urethritis in men, and an infection during pregnancy is associated with preterm delivery, low birth weight, and increased infant mortality^3^. Chronic infection has been implicated as a risk factor for acquisition of human immunodeficiency virus and predisposition to cervical and prostatic cancers^4^. While 5-nitroimidazole antimicrobial agents, such as metronidazole and tinidazole, have been used to treat *T. vaginalis* infections, several studies have reported the emergence of drug-resistant, clinical *T. vaginalis* isolates^5^.

The first draft genome sequence of *T. vaginalis* was published in 2007 as a highly fragmented assembly^6^. The 160 Mb *T. vaginalis* genome is the largest of any unicellular parasite genome currently available and contains ~60,000 predicted protein-coding genes^7^. Considering the large genome size, high repeat copy number, low repeat copy polymorphism, the massive expansion of many gene families, and the retention of multiple copies for almost all genes, it is suggested that the *T. vaginalis* genome has undergone one or more large-scale genome duplication events^6^.

During an infection, *T. vaginalis* must survive a changes in pH, temperature, and iron concentrations^8^. To endure to these adverse conditions, *T. vaginalis* regulates the expression and silencing of various genes at the transcriptional level^9^. Regulation of gene expression is a complex process controlled by several molecular mechanisms, including sequence-specific DNA binding proteins and their cognate DNA regulatory elements, as well as modulation of chromatin structure^10^. One study has shown that *T. vaginalis* uses a metazoan initiator-like element as the sole core promoter element to initiate the transcription of most of its protein-coding genes^11^. This initiator element was found to be specifically recognized by the initiator binding protein IBP39^12^, which interacts with the C-terminal domain of RNA polymerase II^11^. Notwithstanding, other mechanisms by which this parasite regulates transcription have only been partially characterized, and very few DNA regulatory elements and transcription factors have been identified.

Emerging evidence from multiple model organisms has indicated that the modification of histone proteins plays critical roles in gene regulation^13^. Histone modifications (i.e., acetylation and methylation) occur at specific amino acids along the N-terminal tails of core histones, altering chromatin structure and function by changing the charges of nucleosome particles and/or by recruiting protein complexes, either individually or in combination^14^. Studies have shown histone modifications to be associated with transcriptional activation and repression: For example, histone acetylation at lysine residues is generally thought to allow for a more relaxed chromatin state and transcriptional activation, whereas deacetylation of lysine residues facilitates a more compact chromatin state and transcriptional gene silencing by limiting access to transcription machinery^15^. Methylation of histones at lysine residues can both activate and repress gene expression, depending on the position and state thereof^16^.

Acetylation levels are strictly regulated by the concerted activities of histone acetyl transferases (HATs) and histone deacetylases (HDACs)^17^. The HDAC superfamily is grouped into different classes based on sequence similarity and cofactor dependence^18^. In human cells, class I HDACs include HDAC 1–3 and HDAC 8; class II HDACs comprise HDAC 4–7, HDAC9, and HDAC 10; class III HDACs consist of SIRT 1–7; and class IV HDAC comprises only HDAC11. Classes I, II, and IV share a common homology with zinc-dependent yeast Rpd3 or Hda1, while Class III HDACs are homologous to the yeast enzyme silent information regulator 2 (Sir2), which deacetylates lysine residues by consuming NAD^+^. To date, several HDAC inhibitors have been isolated, each with different downstream cellular effects^19^. Importantly, many HDAC inhibitors have been shown to increase the acetylation of core histones, resulting in altered gene expression, and are being investigated as drugs for a range of diseases, including cancers and infectious diseases^19^,^20^.

In this study, we aimed to investigate whether epigenetic chromatin modifications play a role in the modulation of gene expression in *T. vaginalis*. To do so, we first explored the presence of histone modifying enzymes, as well as posttranslational modifications in histone tails, which constitute key components of epigenetic indexing systems. We also examined whether iron-regulated gene expression is subject to epigenetic regulation.

## Results

### Genome-wide transcriptional responses of *T. vaginalis* to histone deacetylase inhibitors

**Figure 1:**
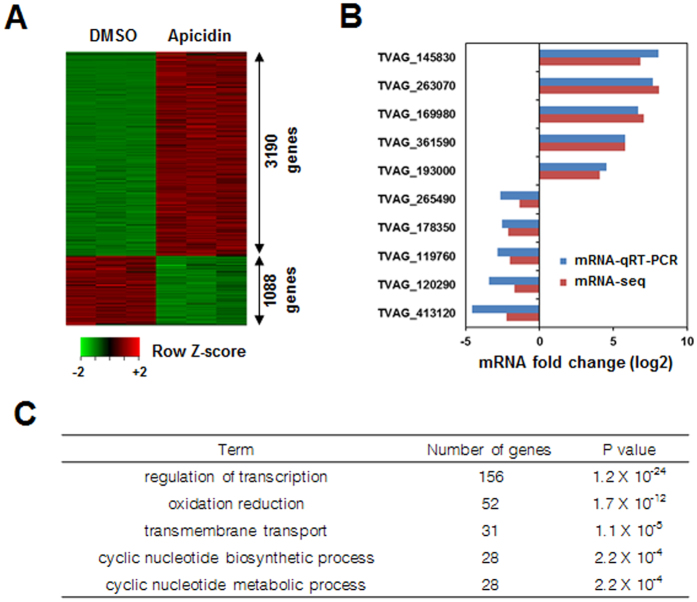
Global transcriptional responses of *T. vaginalis* to apicidin treatment. *T. vaginalis* cells were treated with DMSO (0.1%) or apicidin (70 nM) for 4 hours. RNA samples in each condition were collected and analyzed by RNA-seq. (**A**) Heat map representation of genes differentially expressed upon apicidin treatment. Only significantly changed genes (fold change >2, adjusted p-value < 0.05) are shown. (**B**) qRT-PCR was performed to validate the mRNA targets derived from RNA-seq. (**C**) Gene Ontology (GO) analyses of differentially expressed genes were performed to identify related biological functions.

To delineate the role of histone acetylation in transcriptional regulation, *T. vaginalis* cells were cultured in the presence or absence of apicidin, a class I/II HDAC inhibitor^21^, for 4 hours, after which gene expression profiles were compared by RNA-seq analysis. Genes whose expression varied by a minimum of two-fold following apicidin treatment were considered significantly regulated (adjusted p-value < 0.05). Overall, 4278 genes were found to be differentially regulated by apicidin (Fig. 1A, Supplementary Dataset 1), corresponding to ~7.4% of the 57796 currently predicted *T. vaginalis* genes. The majority of the differentially regulated genes were upregulated in the presence of apicidin (3190 genes upregulated versus 1088 downregulated), indicating that HDAC inhibition acts primarily by promoting the transcription of a select set of genes. The RNA-seq data were validated by quantitative realtime–polymerase chain reaction (qRT–PCR) for several altered genes (Fig. 1B). Functional enrichment analyses of the differentially expressed genes revealed the most significantly enriched pathways to be related with the regulation of transcription or oxidation/reduction (Fig. 1C). In particular, the expression of many regulatory factors, such as multiple MYB transcription factors and the chromatin regulatory protein SIR2, were subject to histone acetylation.

We also explored the impact of another HDAC inhibitor, Trichostatin A (TSA)^22^, on global gene expression profile. Upon treatment therewith, hundreds of genes were differentially regulated, and similar functional groups to those above were affected by treatment with TSA (Supplementary Fig. 1, Supplementary Dataset 2). Importantly, treatment with neither apicidin (70 nM) nor TSA (1 μM) for 4 hours significantly altered cell viability or apoptosis at the chosen concentrations (Supplementary Fig. 2), demonstrating that the changes in global gene expression induced by apicidin or TSA were not caused by toxicity or dying parasites. Taken together, two independent HDAC inhibitors induced profound global transcriptional changes in *T. vaginalis*, suggesting that the regulation of gene expression in *T. vaginalis* may depend on histone acetylation.

### HDAC enzymes in *T. vaginalis*

Next, we analyzed the Trichomonas Genome Database (available from TrichDB at http://trichdb.org/trichdb) to examine whether HDAC enzymes are present in *T. vaginalis*. The *T. vaginalis* genome encodes genes for nine homologues of the Rpd3 family HDACs and eleven homologues of the Sir2 family HDACs (Supplementary Table 1). Additionally, genes for eight homologues of Gcn5 family HATs and twelve homologues of Myst family HATs were identified in *T. vaginalis* genome (Supplementary Table 2). Analysis of RNA-seq data showed that most of these HDAC and HAT genes are indeed expressed at various levels and that some of them are differentially expressed upon treatment with apicidin (Supplementary Fig. 3). Thus, this parasite may possess a rich repertoire of enzymes involved in histone acetylation and deacetylation.

**Figure 2:**
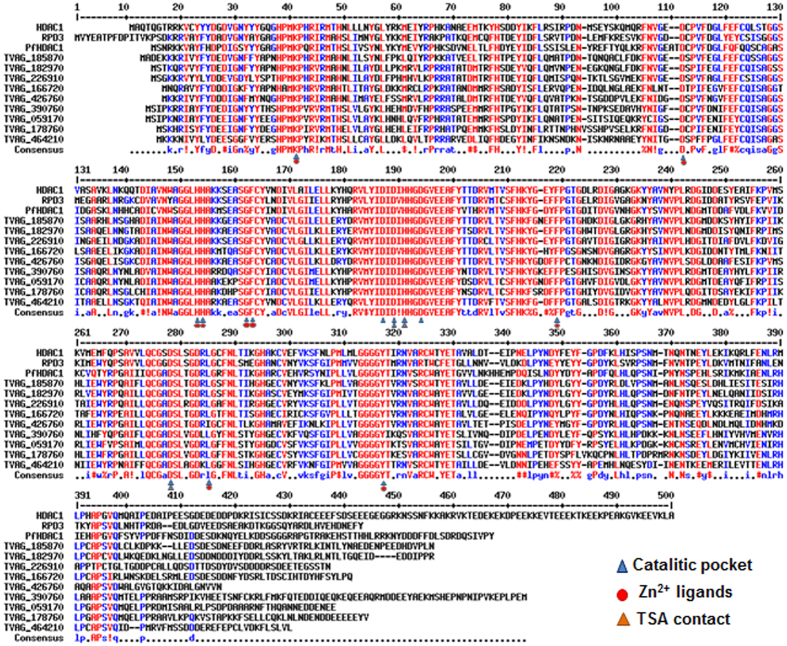
Histone deacetylase enzymes in *T. vaginalis*. Multiple protein sequence alignment of T. vaginalis HDACs with select orthologues. Indicated are residues important for catalytic activity and involved in zinc and TSA binding, based on the crystal structure of related HDACs^24^,^25^. Alignments were performed with Multalin (http://bioinfo.genotoul.fr/multalin/). Gene bank accession numbers: human HDAC1 (NP_004955), *Saccharomyces cerevisiae* Rpd3 (AAB20328.1), and *Plasmodium falciparum* pfHDAC1 (XP_001352127.1).

Since TSA, which elicited changes in global gene expression profiles in *T. vaginalis* (Supplementary Fig. 1), was previously shown to inhibit the activity of Rpd3 family HDACs but not those of the Sir2 family^23^, we focused on nine putative Trichomonas HDACs homologous to Rpd3 and analyzed their phylogenetic relationship with HDACs from other organisms. By comparing amino acid sequences and constructing a phylogenetic tree, we confirmed that all of the nine putative Trichomonas HDACs exhibited close relationships with class I HDACs, including HDAC1, HDAC2, HDAC3, HDAC8, RPD3 and PfHDAC1 (Supplementary Fig. 4). Furthermore, multiple sequence alignment demonstrated a high level of similarity between the nine Trichomonas HDACs and orthologues of human (HDAC1), yeast (Rpd3), and *Plasmodium falciparum* (PfHDAC1) (Fig. 2), confirming these nine Trichomonas HDACs as class I HDACs. Importantly, the amino acid residues in the catalytic pocket that binds TSA and the cofactor Zn2^+^ 24,25 were also present in all of the nine Trichomonas HDACs.

### Inhibition of HDAC induces global histone acetylation in *T. vaginalis*

**Figure 3:**
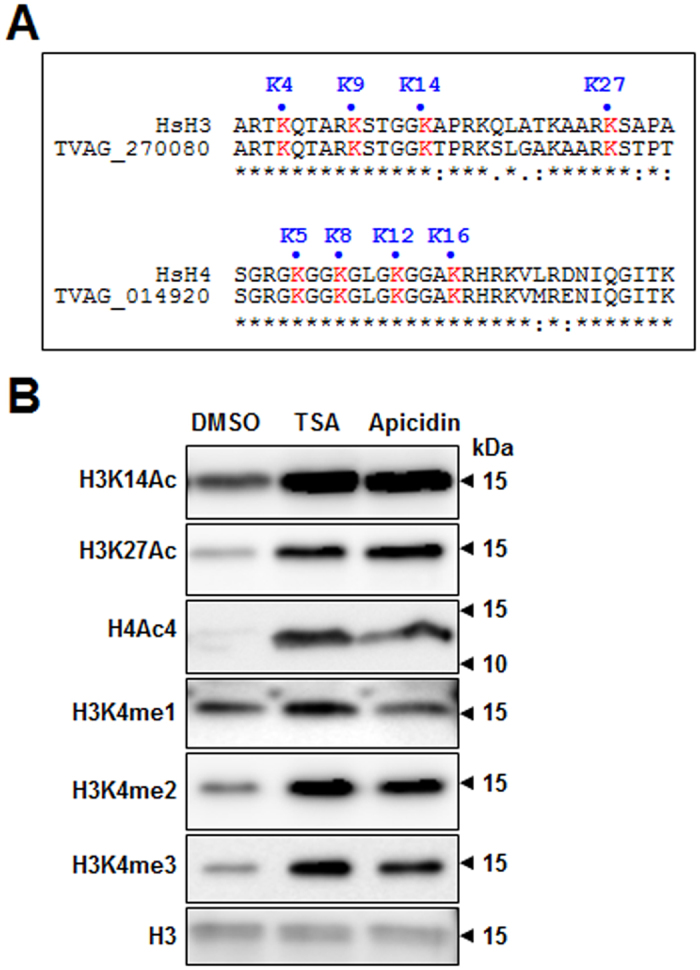
Histone modifications conferred by stimulation with HDAC inhibitors in *T. vaginalis*. (**A**) Identification of epigenetic marks in *T. vaginalis*. Alignment of the H3 and H4 N-termini for *Homo sapiens* (Hs) and *T. vaginalis* (Tv). (*) identical residues; (.) and (:) weakly and strongly similar residues, respectively. (**B**) *T. vaginalis* cells were treated with DMSO (0.1%), TSA (1 μM), or apicidin (70 nM). Protein samples collected at 4 hours post treatment were analyzed by SDS-PAGE. The H3K14Ac, H3K27Ac, H4Ac4, H3K4me1, H3K4me2, and H3K4me3 sites were detected by immunodetection. Total histone H3 was used as a loading control. Molecular weights are shown in kDa. Blots shown have been cropped from full length blot. Full length blots are shown in Supplementary Fig. 9.

The *T. vaginalis* genome contains 20 copies of the core histone H3 and 21 copies of H4. At the amino acid sequence level, these histones are 100% identical; only one H4 gene (*TVAG_100580*) is different^26^. Multiple sequence alignment of the H3 and H4 histones in *T. vaginalis* with those in humans revealed conservation of N-terminal amino acid residues, including lysines at positions 4, 9, 14, and 27 of H3 and lysines at position 5, 8, 12, and 16 of H4 (Fig. 3A). Given that these lysine residues are subject to posttranslational modifications in other metazoan cells^27^, the noted conservation suggests that acetylation or methylation thereof could also occur in the H3 and H4 histones of *T. vaginalis*.

To investigate the mechanism by which HDAC inhibitors alter gene expression in *T. vaginalis*, we examined the effect of TSA and apicidin on the overall levels of three distinct histone acetylations: histone 3 lysine 14 (H3K14Ac), histone 3 lysine 27 (H3K27Ac), and histone 4 lysine residues 5, 8, 12, and 16 (H4Ac4). When we performed immunoblot assays of total cell lysates from *T. vaginalis*, higher levels of histone acetylation were detected in the cells treated with HDAC inhibitors (Fig. 3B). In addition to histone acetylation, the overall levels of mono-, di-, and tri-methylation of histone 3 lysine 4 (H3K4me1, H3K4me2, and H3K4me3), which have been reported to be linked with gene expression in model organisms^16^, were also increased by the HDAC inhibitors (Fig. 3B); the only exception was that apicidin treatment did not increase H3K4me1 levels. These data suggest that acetylation and methylation indeed take place in the core histones H3 and H4 and that these epigenetic modifications might play a role in transcriptional regulation for this pathogen.

### Both H3K4me3 and H3K27Ac are associated with active transcription

**Figure 4:**
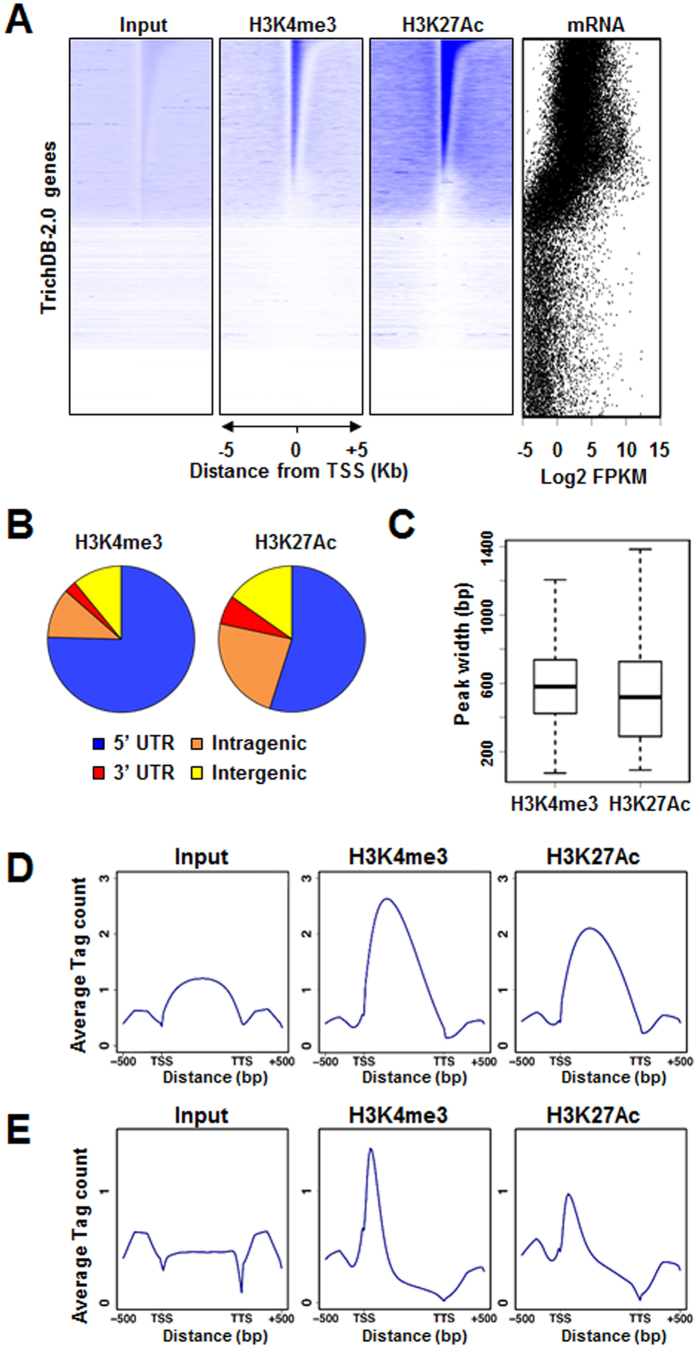
Genome-wide mapping of H3K4me3 and H3K27Ac in mock-treated *T. vaginalis*. (**A**) Density heat map shows coverages for input, H3K4me3, and H3K27Ac across 10 kb centered at the TSS of each gene, along with relative mRNA values. Individual sequences were binned in 25 bp windows (400 bins per sequence), and coverage was computed and plotted as a relative color intensity scale. In all representations, genes were ranked according to their H3K27Ac levels. (**B**) Pie charts present the distribution of H3K4me3 and H3K27Ac peaks among different gene features: 5′UTR, overlapping with TSS (including peaks comprising the whole gene); 3′UTR, overlapping with gene ends; intragenic, inside genes excluding the peaks that overlapped with the 5′- or 3′-ends of the gene; intergenic, upstream and downstream regions of genes. (**C**) Median widths of H3K4me3 and H3K27Ac peaks. (**D** and **E**) Distribution of chromatin input, H3K4me3, and H3K27Ac along gene length in all genes (**D**) or in relatively longer genes (gene size >2 kb) (**E**). TSS: transcription start site, TTS: transcription termination site.

To examine the role of histone modifications in the regulation of gene expression, we investigated the distribution of H3K4me3 and H3K27Ac epigenetic marks along the *T. vaginalis* genome. For this, we conducted chromatin immunoprecipitation in conjunction with high-throughput sequencing (ChIP-seq) using antibodies against H3K4me3 and H3K27Ac (Fig. 4A). ChIP-seq profiles revealed the enrichment of H3K4me3 or H3K27Ac marks in ~25,000 discrete genomic regions. Interestingly, these epigenetic marks were preferentially mapped in 5′ untranslated regions (UTR) and intragenic coding sequences (Fig. 4B). Considering that the mean length of Trichomonas genes are relatively short (928.6 bp)^6^, compared to the average peak widths of these epigenetic marks (595 bp for H3K4me3 and 549 bp for H3K27Ac) (Fig. 4C), we analyzed the distribution of these marks among genes longer than 2 kb and found that both H3K4me3 and H3K27Ac marks were mostly enriched in the 5′-ends of coding sequences (Fig. 4D and E).

**Figure 5:**
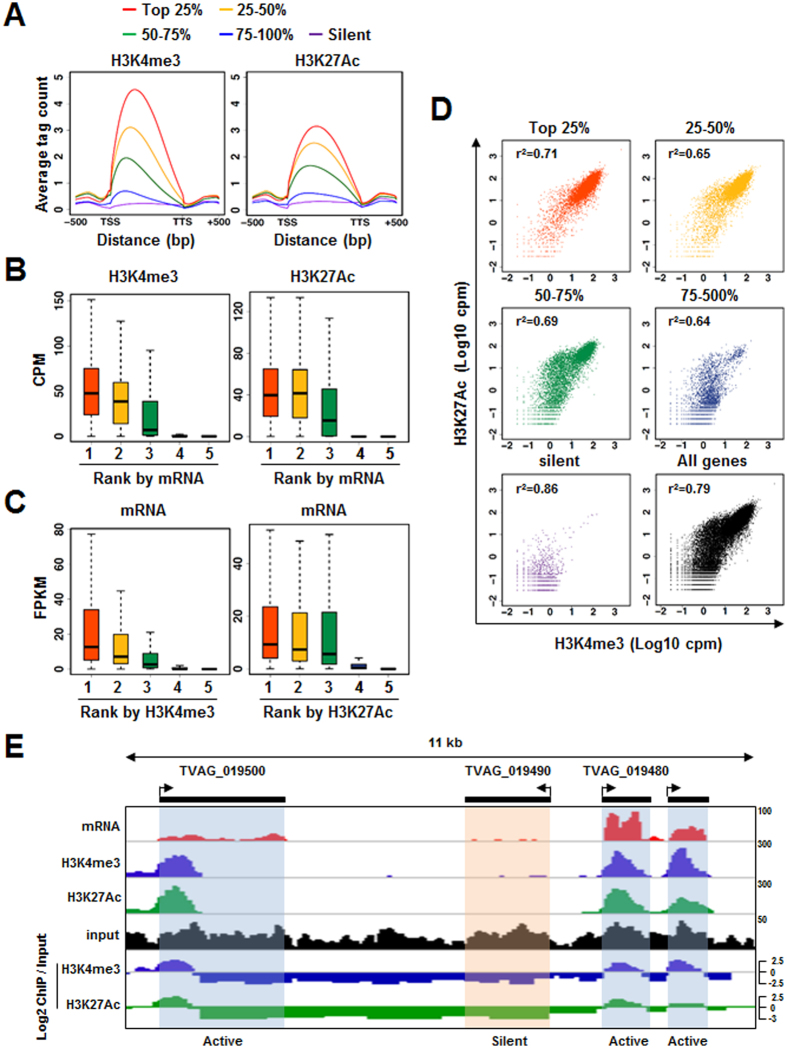
H3K4me3 and H3K27Ac are associated with active transcription in the steady state. Genes were categorized into five ranks (ranks 1–5) according to relative mRNA expression (**A**,**B** and **D**) or relative histone modification levels in the gene body (**C**). Silent genes (rank 5) comprise those with no RNA-seq or ChIP-seq read. (**A**) Distribution of H3K4me3 and H3K27Ac along gene length for genes categorized by mRNA expression. (**B**) Boxplot shows the relative levels of H3K4me3 or H3K27Ac for genes categorized according to mRNA expression, as in (**A**). (**C**) Boxplot shows the relative mRNA expression values of genes categorized according to H3K4me3 and H3K27Ac levels. (**D**) Scatter plot showing H3K4me3 and H3K27Ac levels for genes categorized according to mRNA expression, as in (**A**). Pearson’s correlation coefficient (r^2^) was used to estimate the relationship between H3K4me3 and H3K27Ac levels. (**E**) Genomic snapshot of the *TVAG_019490* locus. Densities of RNA-seq reads and ChIP-seq reads for H3K4me3, H3K27Ac, and input in mock-treated cells are shown.

We also determined the distributions of H3K4me3 and H3K27Ac along gene length for all *T. vaginalis* genes categorized by their relative expressions based on RNA-seq (Supplementary Table 3). Highly transcribed genes (top 50%; ranks 1 and 2) exhibited strong H3K4me3 or H3K27Ac levels in gene coding regions, whereas ~23,000 silent genes, in which mRNA sequencing reads were absent (rank 5), were depleted of these marks (Fig. 5A). As most of the H3K4me3 and H3K27Ac reads, if any, were mapped between transcription start sites (TSS) and transcription termination sites (TTS), we systematically calculated enrichment levels of H3K4me3 and H3K27Ac across the gene body (from TSS to TTS) for all *T. vaginalis* genes and compared these results with gene expression levels. Compared to silent genes (rank 5) or lowly expressed genes (rank 4), much higher levels of H3K4me3 and H3K27Ac were detected in actively expressed genes (ranks 1 and 2) (Fig. 5B). We also compared levels of relative mRNA expression between genes categorized based on ChIP-seq reads (Supplementary Table 3). Therein, genes exhibiting higher levels of H3K4me3 or H3K27Ac (ranks 1, 2, and 3) were more actively transcribed than those with lower levels of H3K4me3 or H3K27Ac (rank 4). Transcription was completely repressed for more than 25,000 genes in which there were no H3K4me3 or H3K27Ac reads (rank 5) (Fig. 5C). Interestingly, enrichment of H3K4me3 marks showed a significantly high correlation with enrichment of H3K27Ac marks (Fig. 5D). In particular, H3K4me3 and H3K27Ac marks were simultaneously enriched in the highly transcribed genes (ranks 1 and 2), while few of these marks were observed in silent genes (rank 5) (Fig. 5D). When we analyzed independent biological replicates of ChIP-seq data for H3K4me and H3K27Ac, similar correlation was demonstrated (data not shown). Taken together, these data demonstrated that simultaneous enrichment of two epigenetic marks, H3K4me3 and H3K27Ac, is positively correlated with active gene expression in the steady state of transcription for *T. vaginalis*. One of the best examples of these positive correlations is highlighted in Fig. 5E: An actively transcribed gene, *TVAG_019500*, exhibits strong enrichment of both H3K4me3 and H3K27Ac in the 5′-ends of coding sequences, while these chromatin marks were depleted in the silent gene *TVAG_019490* (Fig. 5E).

### HDAC inhibition elicits genome-wide changes in H3K4me3 and H3K27Ac levels

**Figure 6:**
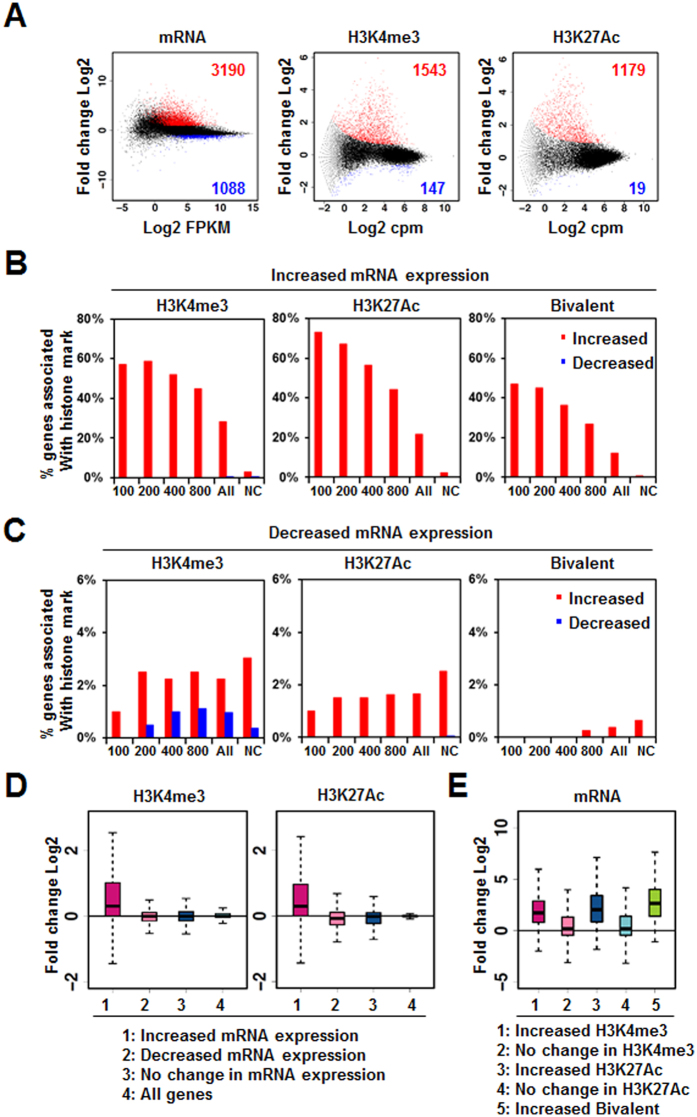
Genome-wide changes in H3K4me3 and H3K27Ac levels upon apicidin treatment. (**A**) MA plots show fold changes and relative read concentrations for mRNA, H3K4me3, and H3K27Ac. Significantly changed genes (FDR < 0.05) are indicated in red (upregulated in apicidin-treated cells) or blue (downregulated in apicidin-treated cells). (**B** and **C**) Correlations between increased (**B**) or decreased (**C**) mRNA expression are shown for increased (red) or decreased (blue) levels of H3K4me3, K3K27Ac, and bivalent marks. Bivalent marks include H3K4me3 and H3K27Ac. Only regions with preexisting H3K4me3 or H3K27Ac (cpm > 0) were included in the analyses. Genes were grouped (x-axis) according to degrees of the up- or downregulation observed in the RNA-seq analysis, as a cumulative rank (e.g., top 100 genes, top 200 genes, and so on, where the largest “all” category represents the entire up- or downregulated gene set and “NC” represents no change, adjusted p < 0.05). Correlation is reported as the percentage of genes in each rank associated with changes in each chromatin mark in the gene body. (**D**) Box plot shows the relative fold changes in the levels of H3K4me3 or H3K27Ac for genes categorized by changes in mRNA expression. (**E**) Box plot shows the relative fold changes in mRNA expression for genes categorized according to changes in the indicated chromatin marks.

Although immunoblot analysis indicated that HDAC inhibitors increased the overall levels of H3K4me3 and H3K27Ac, we wished to identify which chromatin regions are under the control of HDAC. Thus, we evaluated the effect of apicidin on the genome-wide distribution of H3K4me3 and H3K27Ac via ChIP-seq analysis (Fig. 6A). Upon treatment with apicidin, H3K4me3 and H3K27Ac levels increased significantly in 1543 and 1179 genes, respectively (Fig. 6A, Supplementary Dataset 3 and 4). Increases in H3K4me3 and H3K27Ac were mostly identified in genes with low levels of these marks prior to apicidin stimulation (Fig. 6A), suggesting that the *T. vaginalis* genome is subject to histone acetylation by HDAC inhibitor at regions previously hypoacetylated. In contrast, few genes showed significant decreases in H3K4me3 and H3K27Ac (147 and 19 genes, respectively) after exposure to apicidin. Interestingly, simultaneous increases in both H3K4me3 and H3K27Ac were detected in 497 genes upon apicidin treatment, while no simultaneous decreases in these marks was detected in any of the genes. Thus, we determined that apicidin treatment is much more likely to induce increases in H3K4me3 and H3K27Ac than decreases in these marks.

We then sought to determine whether changes in gene expression induced by apicidin treatment are accompanied by histone modification changes. We found that 57%, 73%, and 47% of the top 100 most upregulated genes were associated with increased levels of H3K4me3, H3K27Ac, and both marks simultaneously (bivalent marks), respectively (Fig. 6B). In contrast, only 3.0%, 2.5%, and 0.6% of the genes with no change in expression were associated with increased levels of H3K4me3, H3K27Ac, and bivalent marks, respectively (Fig. 6B). Moreover, almost none of the upregulated genes showed decreased levels of these epigenetic marks (Fig. 6B). These results suggested that genes exhibiting large increases in expression are more likely to be associated with increased H3K4me3 and/or H3K27Ac levels. In regards to downregulated genes, only a few (less than 3%) showed increases in H3K4me3 and H3K27Ac levels; the statistical strength of this relationship, however, was not greater than that for genes with no change in expression (Fig. 6C). Decreased H3K27Ac was not identified in any of the downregulated genes, although a slight association was noted between downregulated genes and decreased H3K4me3 (Fig. 6C). Thus, decreased mRNA expression induced by apicidin treatment reflected little or no correlation with changes in H3K4me3, H3K27Ac, and bivalent marks.

We then compared changes in histone modification for genes categorized according to changes in mRNA expression, and increased levels of H3K4me3 and H3K27Ac were evident only in genes with increased mRNA expression (Fig. 6D). We also compared relative levels of mRNA expression for genes categorized according to changes in histone modification, and mRNA expression was induced only for genes with increased levels of H3K4me3, H3K27Ac, or both marks (Fig. 6E).

The correlations of histone modification changes with gene expression were also determined using Fisher’s exact test (Table 1). Increased mRNA expression was more likely to occur with increased H3K4me3, H3K27Ac, and bivalent mark with odds ratios of 18.228, 19.261, and 41.941, respectively. Meanwhile, however, reduced gene expression demonstrated poor correlation with these histone modifications, although decreased mRNA expression showed modest positive correlation with decreased H3K4me3, with an odds ratio of 3.252.

The impact of HDAC on the genome-wide changes in histone modifications was further investigated using another HDAC inhibitor, TSA. Similar to apicidin, TSA treatment was much more likely to induce increases in H3K4me3 and H3K27Ac than decreases in these marks (Supplementary Fig. 5A, Supplementary Dataset 5 and 6). Moreover, increases in H3K4me3 and H3K27Ac levels were evident only in the genes with increased mRNA expression (Supplementary Fig. 5B), and induced mRNA expression was observed only in genes with increased levels of H3K4me3, H3K27Ac, or both marks (Supplementary Fig. 5C).

We validated the results of our genome-wide analysis through qRT-PCR and ChIP-qPCR assays. Induced expression of *TVAG_030540* and *TVAG_169980* upon treatment with either apicidin or TSA coincided with increases in H3K4me3 and H3K27Ac (Supplementary Fig. 6). Taken together, our results suggested that HDAC inhibitors, such as apicidin and TSA, are more likely to cause hyperacetylation than hypoacetylation and that increased histone acetylation may drive increases in H3K4me3 levels resulting in gene expression.

### Histone modification in iron-regulated genes

**Figure 7:**
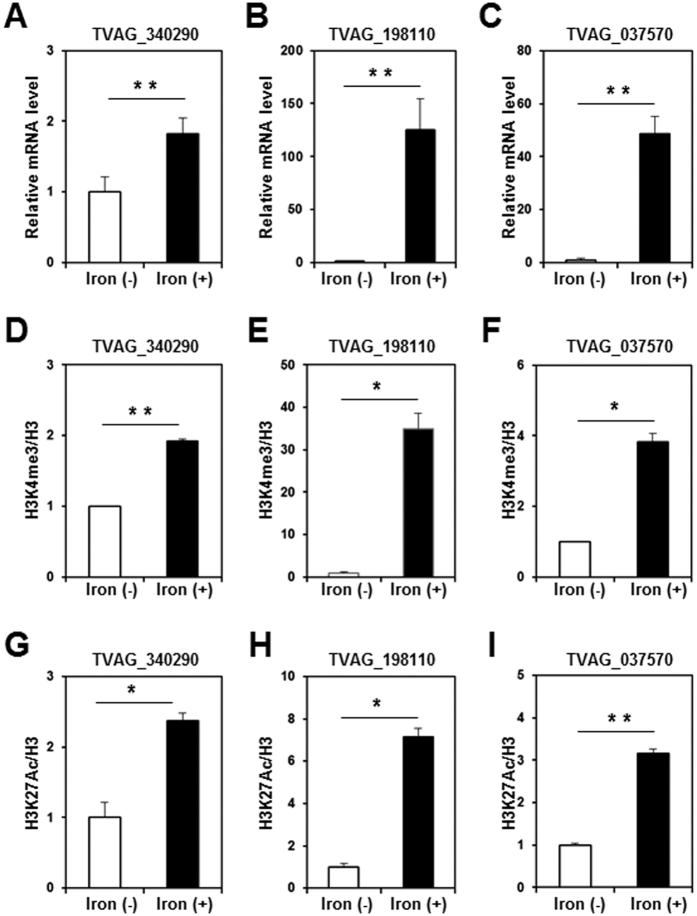
Histone modification in iron-regulated genes of *T. vaginalis*. *T. vaginalis* cells were grown in an iron-restricted or iron-rich condition for 18 hours. (**A–C**) The RNA levels of *TVAG_340290* (**A**), *TVAG_198110* (**B**), and *TVAG_037570* (**C**) were analyzed by qRT-PCR. (**D**–**I**) The enrichment of H3K4me3 (**D**–**F**) and H3K27Ac (**G**–**I**) in *TVAG_340290* (**D** and **G**), *TVAG_198110* (**E** and **H**), and *TVAG_037570* (**F** and **I**) was analyzed by ChIP-qPCR. Data represent at least three independent experiments. Error bars indicate SD. *P < 0.05; **P < 0.01.

Iron is an essential nutrient for Trichomonas and plays a pivotal role in the establishment of infection, proliferation, and virulence^9^,^28^,^29^. Since the availability of iron can vary greatly in the human vagina, where *T. vaginalis* inhabits, this parasite may be able to adapt to this hostile host environment and maintain iron homeostasis by modulating the expression of multiple genes. Iron-dependent gene regulation at the transcriptional level has been well described in the ap65-1 gene that encodes a 65 kDa surface protein with sequence homology to hydrogenosomal malic enzyme^30^,^31^,^32^,^33^,^34^,^35^. Consistent with this previous report, we confirmed increased expression of *TVAG_340290*, encoding the ap65-1 gene, in an iron-rich condition, compared to an iron-restricted condition (Fig. 7A). In addition to the ap65-1 gene, previous transcriptome analysis identified many genes to be differentially expressed according to varying iron concentrations^36^. Among the reported iron-regulated genes, we selected two (*TVAG_198110* and *TVAG_037570*) for qRT-PCR analysis to validate the increased expression thereof according to iron availability (Fig. 7B and C). Interestingly, ChIP-qPCR analysis revealed greater amounts of H3K4me3 and H3K27Ac in these iron-regulated genes in the iron-rich condition than in the iron-restricted condition (Fig. 7D–I). These results indicated that changes in histone modification, such as H3K4me3 and H3K27Ac, are positively associated with dynamic gene regulation in response to changing physiological conditions.

## Discussion

In the present study, we completed the first mapping of the epigenome of *T. vaginalis* and identified H3K4me3 and H3K27Ac as global epigenetic marks for active gene expression in the steady state, as well as during dynamic transcriptional changes (Supplementary Fig. 7). Chromatins from eukaryotic cell types typically display a relatively narrow and promoter-specific localization of H3K4me3, primarily at promoters of actively transcribed genes^37^. H3K27Ac, first identified in yeast^38^ and later in mouse and human cells^39^, is highly enriched at promoter regions of transcriptionally active genes, as well as at nucleosomes flanking enhancer elements^40^,^41^. Our epigenome profiling demonstrated that H3K4me3 and H3K27Ac are distributed along the genome of *T. vaginalis*, closely resembling that of classic model organisms: both of these epigenetic marks were preferentially enriched at the 5′-ends of the coding regions of actively transcribed genes. In contrast, around 23,000 silent genes on RNA-seq analysis were completely depleted of these marks, further highlighting that H3K4me3 and H3K27Ac are landmarks of active gene expression in *T. vaginalis*.

The H3K4me3 and H3K27Ac marks have been shown to be recognized by “reader proteins” in other eukaryotes and to be important in chromatin remodeling and recruitment of transcriptional machinery. For example, the H3K4me3 mark is reportedly recognized by PHD finger proteins that help initiate transcription, such as the core transcription factor TFIID and the NURF chromatin remodeling complex^42^,^43^. Also, the H3K4me3 mark has been found to affect transcription elongation via the chromodomains of mammalian CHD1, which recruit factors, such as FACT and the PAF complex^44^. Meanwhile, H3K27Ac might be recognized by bromodomain-containing proteins required for transcription^45^. H3K27Ac may also prevent the repressive trimethylation of the same lysine residue, as acetylation and trimethylation of H3K27 (H3K27me3) are considered mutually exclusive in model organisms^46^. While H3K27me3 is present in Drosophila and mammals, it is absent in simple model organisms, such as *S. cerevisiae* and *P. falciparum*^47^,^48^. As the presence of repressive marks, such as H3K27me3, has yet to be determined in *T. vaginalis*, future studies thereon are warranted.

Our epigenome profiling revealed that loci with high levels of H3K27Ac also display high levels of H3K4me3 in *T. vaginalis*. The coincident presence of these marks in actively transcribed genes may exert a cumulative effect on the recruitment of transcription factors and chromatin modifying enzymes. Cross-talk between these epigenetic marks is also a possibility and has been previously proposed in several model organisms. For example, specific recognition of H3K4me3 by the PHD finger domain of ING4, a subunit of the HBO1 HAT complex, increased HBO1 acetylation activity on H3 tails and drove H3 acetylation at ING4 target promoters^49^. Identification of “reader proteins” that recognize H3K4me3 and H3K27Ac would help improve our understanding of how these histone modifications contribute to gene regulation in *T. vaginalis*.

Increasing evidence implicates epigenetic gene regulation in the adaptation, survival, and virulence of protozoan parasites^50^,^51^,^52^,^53^. The most comprehensive epigenomic studies in parasites have been performed in *P. falciparum*. When these parasites progress to schizont stages, the classic gene-activation marks H3K4me2, H3K4me3, H3K9Ac, H3K14Ac, and histone H4Ac are found at the 5′ region of actively transcribed genes, as in other eukaryotes^54^. However, H3K4me3 and H3K9Ac are spread evenly across both active and inactive genes and do not correlate with transcriptional activity in synchronized ring stage *P. falciparum*^54^. Additionally, H3K27Ac levels in *P. falciparum* are very low and do not show any correlation with gene activity^48^, contrary to our observation in *T. vaginalis*. Another protozoan parasite model, *T. gondii*, shows euchromatin marks of H4Ac, H3K9Ac, and H3K4me3, which colocalize and mark the promoters of actively transcribed genes^55^. However, stage-specific bradyzoite and sporozoite promoters are not enriched with these euchromatin marks^55^.

Upon transcriptome and epigenome profiling, we found the majority of *T. vaginalis* genes that were responsive to the HDAC inhibitors apicidin and TSA to be upregulated, which was positively associated with increased H3K4me3 and H3K27Ac levels, although many genes were downregulated. Considering that the HDAC inhibitors were more likely to induce hyperacetylation than hypoacetylation and that decreased gene expression was not associated with alteration of histone acetylation levels, any repressed gene expression mediated by HDAC inhibitors may have been indirect and occurred via the action of a repressor positively regulated by increased histone acetylation. Indeed, our RNA-seq data demonstrated that the expressions of many transcription factors and chromatin modifying enzymes were modulated by HDAC inhibitors, which may have caused the repression of their target genes. Alternatively, HDACs of *T. vaginalis* may regulate gene expression independent of histone proteins.

According to recent transcriptomic and proteomic analyses, iron modulates the expression of important metabolic enzymes and several virulence factors, thereby affecting the virulence properties of *T. vaginalis*. Although some cysteine proteases are reportedly regulated at the posttranscriptional level by an iron-responsive element/iron response protein-like system^56^, iron-dependent gene regulation at the transcriptional level has been described only for the *ap65*-*1* gene. The *ap65*-*1* gene has an iron-inducible core promoter and several regulatory elements that are recognized by three Myb transcription factors, TvMyb1, TvMyb2, and TvMyb3^30^,^31^,^32^,^33^,^34^,^35^. Although the presence of Myb recognition elements are shared by other genes, no direct evidence on the role of this regulatory element in iron-dependent gene transcription has been provided for other genes^56^. In the present study, we reported for the first time that histone modifications contribute to iron-dependent transcriptional regulation in *T. vaginalis*. The expression of iron-regulated genes, including the aforementioned *ap65*-*1 (TVAG_340290*), as well as two other genes (*TVAG_198110* and *TVAG_037570*), was positively associated with H3K4me3 and H4K27Ac levels at the 5′-ends of coding sequences. These results suggested that this parasite depends on a histone modification system to allocate a functional role to certain DNA sequences that in turn control iron-dependent gene expression.

Taken together, we provide the first evidence that suggest that posttranslational modifications of histones play an essential role in transcriptional regulation in *T. vaginalis*. The high-resolution genome-wide profiles of histone modifications that we generated in this study can be used as a reference epigenome of *T. vaginalis*, with which to study control mechanisms of gene expression involved in the invasion, metabolism, immune evasion, and other essential processes exhibited by this parasite. Doing so might uncover novel targets for therapeutic intervention.

## Methods

### Cultivation of *T. vaginalis*

The T016 strain of *T. vaginalis*^57^ was axenically subcultivated at 37 °C in Diamond’s trypticase–yeast extract–maltose (TYM) medium with 10% heat-inactivated horse serum (Gibco) and 0.5% penicillin/streptomycin (Gibco). *T. vaginalis* cells were stimulated with 70 nM apicidin (Sigma) or 1μM TSA (Sigma) for 4 hours for RNA-seq and ChIP-seq. Iron-rich medium and iron-restricted medium were supplemented with 250 μM ammonium iron (II) sulfate (Sigma) and 50 μM 2′-2-bipyridyl (Sigma), respectively.

### Quantitative real-time polymerase chain reaction (qRT-PCR)

Total RNA from *T. vaginalis* cells was isolated with the Hybrid-R Total RNA kit (GeneAll Biotechnology). cDNA was synthesized using PrimeScript™ RT Master Mix (Takara Bio). Quantitative real-time PCR was performed with the ABI StepOnePlus real-time PCR system (Applied Biosystems), monitoring the synthesis of double-stranded DNA using SYBR Green (Qiagen). For each sample, duplicate test reactions were analyzed for the expression of the gene of interest, and results were normalized to β-tubulin mRNA. The sequences of the primers are listed in Supplementary Table 4.

### Library construction and RNA sequencing

We generated three biological replicates of RNA-seq data in each condition, except in cells treated with TSA (Supplementary Table 5). The RNA sequencing (RNA-seq) library was prepared using a TruSeq RNA Sample Prep Kit (Illumina). The library was sequenced using an Illumina NextSeq 500 system (Illumina) to generate 76 bp paired-end reads. Reads were quality-trimmed and filtered using an NGS QC Toolkit v2.3^58^ to remove reads with low-quality bases (quality score >20). Genomic scaffolds of *T. vaginalis*, sequences of annotated genes, and genomic features were downloaded from TrichDB v2.0 (http://trichdb.org/trichdb/); repeated genes were discarded. High-quality reads were mapped to the *T. vaginalis* genome using RSEM with Bowtie2 v2.0.0-beta7^59^. The expression level of each transcript was quantified as FPKM (fragments per kilobase of exon per million fragments mapped), and the EBSeq package^60^ was used to select differentially expressed genes. The RNA-seq data were visualized using the Integrative Genomics Viewer^61^. Gene Ontology analysis was conducted with DAVID Bioinformatics Resources^62^.

### Western blot analysis

For western blotting of total lysates, cells were lysed in 100 μL of cell lysis buffer containing 50 mM Tris-HCl (pH 7.5), 150 mM NaCl, 1% nonyl phenoxypolyethoxylethanol, 1 mM ethylenediaminetetraacetic acid, 5% glycerol, and protease inhibitor cocktail (Sigma). Whole cell lysates were resolved on sodium dodecyl sulfate-polyacrylamide (SDS-PAGE) gels and transferred onto a polyvinylidene fluoride membrane. After blocking with 5% skim milk, the membrane was incubated with antibodies, followed by incubation with horseradish peroxidase-conjugated secondary antibody. Target proteins were visualized using SuperSignal West Pico Chemiluminescent Substrate (Pierce) and ImageQuant LAS 4000 (GE Healthcare). Antibodies against H3K14Ac and H4Ac4 were obtained from Millipore, and antibodies against H3K27Ac, H3K4me1, H3K4me2, H3K4me3, and H3 were purchased from Abcam.

### Sequence Alignment and phylogenetic analysis

Homologues of yeast HDAC and HAT genes were identified using basic local alignment search tool (BLAST) queries of the Trichomonas Genome Database (http://TrichDB.org)^63^. Multiple protein sequence alignment of T. vaginalis HDACs were performed with Multalin (http://bioinfo.genotoul.fr/multalin/)^64^. To identify the phylogenetic positions of the TvHDACs, the predicted amino acid sequences of the putative TvHDACs were aligned with HDACs from other organisms by ClustalW, and data were subjected to phylogenetic analysis by UPGMA using MEGA software, version 7.0.18^65^. Numbers at the branch nodes display branch lengths.

### Chromatin immunoprecipitation (ChIP)

ChIP assays were performed as described previously^66^ with minor modifications. Briefly, *T. vaginalis* cells were cross-linked with 1% formaldehyde, incubated in swelling buffer (25 mM HEPES, pH 7.9, 1.5 mM MgCl_2_, 10 mM KCl, 0.1% NP40, 1 mM DTT), and subjected to sonication in Buffer A (10 mM Tris-HCl pH 8.0, 2 mM EDTA, 0.2% SDS) using a Bioruptor sonication device (Diagenode). Chromatin samples were diluted with Buffer B (10 mM Tris-HCl pH 8.0, 2% Triton X-100, 280 mM NaCl, 0.2% deoxycholate) and immunoprecipitated with antibodies specific for H3K4me3 (Abcam) and H3K27Ac (Abcam). Chromatin-antibody complexes were pulled down by Protein A/G Dynabeads (Invitrogen). After treatment with proteinase K to remove protein and reverse the cross-links, the amounts of selected DNA sequences were assessed by real time PCR. The sequences of the primers are listed in Supplementary Table 4.

### Massive parallel sequencing for ChIP

We generated two biological replicates of ChIP-seq data for H3K4me and H3K27Ac in each condition, except for cells treated with TSA (Supplementary Table 6). For ChIP sequencing, genomic libraries were generated using the TruSeq ChIP Sample Prep Kit (Illumina) from input and chromatin-immunoprecipitated DNA with an average chromatin size of 200–300 bp. The libraries were sequenced using an Illumina NextSeq 500 system to generate 76 bp single-end reads. Reads were quality-trimmed and filtered using an NGS QC Toolkit v2.3^58^ to remove reads with low-quality bases (quality score >20). High-quality reads were mapped to the *T. vaginalis* genome using Bowtie2 v2.0.0-beta7^67^ with the option: bowtie2 –mp 1,1 –np 1 –score-min L,0,-0.1. Read counts from the TSS to TTS were calculated for each gene using HTSeq v0.6.1 [6] and converted to log2 cpm (count per million) values using the Bioconductor package edgeR v3.6.8^68^, which was also used to select genes with differential enrichment of each histone modification. To eliminate biases between libraries, normalization was performed using the trimmed mean of M-values^69^. ChIP-seq replicates were evaluated with cross-correlation analysis using ggplot2 in R package (Supplementary Fig. 8)^70^. Heat map plots for the indicated histone modifications were determined as counts per million in 25 bp bins in the 10 kb region surrounding the TSS using seqMINER^71^. Peak calling from the ChIP-seq data was performed using a HOMER package with default parameters and chromatin input sample as a control^72^. Average profile plots and box plots were generated using R package. ChIP-seq data were visualized using the Integrative Genomics Viewer^61^.

### Data access

The files generated in the RNA-seq and ChIP-seq experiments are available at the Gene Expression Omnibus (GEO) database (https://www.ncbi.nlm.nih.gov/geo/query/acc.cgi?token=mhgdscmybdeprup&acc=GSE89662) with the accession number GSE89662.

### Statistics

Data were analyzed with an unpaired Student’s two-tailed t-test.

## Additional Information

**How to cite this article**: Song, M.-J. *et al*. Epigenome mapping highlights chromatin-mediated gene regulation in the protozoan parasite *Trichomonas vaginalis*. *Sci. Rep.* **7**, 45365; doi: 10.1038/srep45365 (2017).

**Publisher's note:** Springer Nature remains neutral with regard to jurisdictional claims in published maps and institutional affiliations.

## Supplementary Material

Supplementary Information

Supplementary Dataset 1

Supplementary Dataset 2

Supplementary Dataset 3

Supplementary Dataset 4

Supplementary Dataset 5

Supplementary Dataset 6

## Figures and Tables

**Table 1:** Statistical analysis of histone modification and gene expression in apicidin-treated *T. vaginalis*.

mRNA	Histone	p-value	Odds ratio
Increased	↑ H3K4me3	<2.2 × 10^−16^	18.228
	↑ H3K27ac	<2.2 × 10^−16^	19.261
	↑ bivalent	<2.2 × 10^−16^	41.941
	↓ H3K4me3	0.011	2.096
	↓ H3K27ac	0.019	3.671
	↓ bivalent	1.000	0.000
Decreased	↑ H3K4me3	0.484	1.165
	↑ H3K27ac	0.325	1.270
	↑ bivalent	0.562	1.268
	↓ H3K4me3	0.002	3.252
	↓ H3K27ac	1.000	0.000
	↓ bivalent	1.000	0.000
