# Supplementary material for: Epigenome mapping highlights chromatin-mediated gene regulation in the protozoan parasite Trichomonas vaginalis
Source: Sci Rep. 2017 Mar 27;7:45365. doi: 10.1038/srep45365 (PMC5366954; doi:10.1038/srep45365)
Supplement: Supplementary Information [file srep45365-s1.pdf]

Epigenome mapping highlights chromatin-mediated gene regulation in the protozoan parasite *Trichomonas vaginalis*

Min-Ji Song[1,2], Mikyoung Kim[1], Yeeun Choi[1,3], Myung-hee Yi[1], Juri Kim[1], Soon-Jung Park[1], Tai-Soon Yong[1], and Hyoung-Pyo Kim[1,2,3,*]

[1]Department of Environmental Medical Biology, Institute of Tropical Medicine, Yonsei University College of Medicine, Seoul, 03722, Korea

[2]Graduate Program of Nano Science and Technology, Yonsei University College of Medicine, Seoul, 03722, Korea

[3]BK21 PLUS Project for Medical Science, Yonsei University College of Medicine, Seoul, 03722, Korea

*Corresponding. kimhp@yuhs.ac

Supplementary Methods

Cell viability assay

T. vaginalis cells were seeded at a density of 4×10^5 per 5 ml of medium and exposed to apicidin or TSA for 4 hours at the indicated concentrations. Viability was measured by Trypan blue (Invitrogen) exclusion analysis.

Apoptosis assay

Approximately 5×10^5 *T. vaginalis* cells were washed twice with phosphate buffered saline and resuspended in 1× binding buffer (10 mM HEPES/NaOH [pH 7.4], 140 mM NaCl, and 2.5 mM CaCl2). Afterwards, cells were incubated with 5 µl of Annexin V-APC (e-Bioscience) and 10 µl of 50 µg/ml propidium iodide (Sigma) for 15 min at room temperature and then analyzed by flow cytometry using FACS Verse (BD Biosciences)

Supplementary Figure Legends

Supplementary Figure 1. Global transcriptional response of *T. vaginalis* to TSA treatment. *T. vaginalis* cells were treated with DMSO (0.1%) or TSA (1 μM) for 4 hours. RNA samples in each condition were collected and analyzed by RNA-seq. (A) Scatter plot shows genes differentially expressed upon TSA treatment. Significantly changed genes (fold change > 2, adjusted p-value < 0.05) are indicated in red (upregulated in TSA-treated cells) or blue (downregulated in TSA-treated cells). (B) Quantitative real-time PCR (qRT-PCR) was used to validate the mRNA targets derived from RNA-seq, analyzing transcript levels for genes significantly upregulated or downregulated. (C) Gene Ontology (GO) analyses of differentially expressed genes were performed to identify related biological functions. (D) Venn diagram identifying the common and exclusively regulated genes by apicidine or TSA (fold change > 2, adjusted p-value < 0.05). The number of biological replicates for each condition was; DMSO, 3; apicidin, 3; and TSA, 1.

Supplementary Figure 2. The effect of TSA and apicidin on the viability and apoptosis of *T. vaginalis*. (A and B) *T. vaginalis* cells were mock-treated or treated with different concentrations of apicidin (A) or TSA (B) for 4 hours, and cell viability was assessed by Trypan blue staining. (C and D) *T. vaginalis* cells were mock-treated or treated with apicidin (C) or TSA (D) for 4 hours, and cell apoptosis was assessed by flow cytometry.

Supplementary Figure 3. Putative genes encoding histone acetyltransferase and histone deacetylase in *T. vaginalis*. Expression of putative Rpd3 HDAC superfamily (A), Sir2 HDAC superfamily (B), Gcn5 HAT superfamily (C), and Myst HAT superfamily (D) was analyzed by RNA-seq data.

Supplementary Figure 4. Phylogenetic comparison of TvHDAC proteins. The predicted amino acid sequences of the putative TvHDACs were aligned with HDACs from other organisms by ClustalW, and data were submitted to phylogenetic analysis by UPGMA using MEGA version 7.0.18. Protein sequences used for this analysis included HDAC1 (NP_004955), HDAC2 (NP_001578.3), HDAC3 (NP_003874.2), HDAC4 (NP_006028.2), HDAC5 (NP_005465.2), HDAC6 (AAH69243.1), HDAC7 (NP_001091886.1), HDAC8 (NP_060956.1), HDAC9 (NP_478056.1), HDAC10 (NP_114408.3), and HDAC11 (NP_079103.2) for *Homo sapiens*; PfHDAC1 (XP_001352127.1) for *Plasmodium falciparum*; and RPD3 (AAB20328.1) for *Saccharomyces cerevisiae*. Numbers at the branch nodes display branch lengths.

Supplementary Figure 5. Genome-wide changes in H3K4me3 and H3K27Ac upon TSA treatment. (A) MA plots show fold changes and relative read concentrations for mRNA, H3K4me3, and H3K27Ac. Significantly changed genes (FDR < 0.05) are indicated in red (upregulated in TSA-treated cells) or blue (downregulated in TSA-treated cells). (B) Box plot showing relative fold changes in levels of H3K4me3 or H3K27Ac for genes categorized by changes in mRNA expression. (C) Box plot showing relative fold changes in mRNA expression for genes categorized by changes in the indicated chromatin marks. (D and E) Venn diagram showing the number of overlapping H3K4me3-enriched genes (D) or H3K27Ac-enriched genes (E) between apicidin and TSA (fold change > 1.5, adjusted p-value < 0.05).

Supplementary Figure 6. Validation of RNA-seq and ChIP-seq data. (A) Genomic snapshot of the *TVAG_030540* and *TVAG_169980* loci. Density of RNA-seq reads and ChIP-

seq reads for H3K4me3, H3K27me3, H3, and input in *T. vaginalis* cells treated with DMSO, apicidin, and TSA. (B) The RNA levels of *TVAG_030540* and *TVAG_169980* were analyzed by qRT-PCR. Enrichment of H3K4me3 and H3K27Ac in the gene bodies of *TVAG_030540* and *TVAG_169980* were analyzed by ChIP-qPCR. Data represent at least three independent experiments. Error bars indicate SEM. *, P<0.05; **, P<0.01; ***, P<0.001.

Supplementary Figure 7. Genomic snapshot of the *TVAG_019490* locus. Densities of RNA-seq reads and ChIP-seq reads for H3K4me3, H3K27Ac, and input in T. vaginalis cells treated with DMSO, apicidin, and TSA are shown.

Supplementary Figure 8. Heat map and hierarchical dendrogram depicting the cross-correlation matrix across the whole ChIP-seq dataset.

Supplementary Figure 9. Histone modifications conferred by stimulation with HDAC inhibitors in *T. vaginalis*. *T. vaginalis* cells were treated with DMSO (0.1%), TSA (1 μM), or apicidin (70 nM). Protein samples collected at 4 hours post treatment were analyzed by SDS-PAGE. The H3K14Ac, H3K27Ac, H4Ac4, H3K4me1, H3K4me2, and H3K4me3 sites were detected by immunodetection. Total histone H3 was used as a loading control. Molecular weights are shown in kDa.

Supplementary Figure 1

A

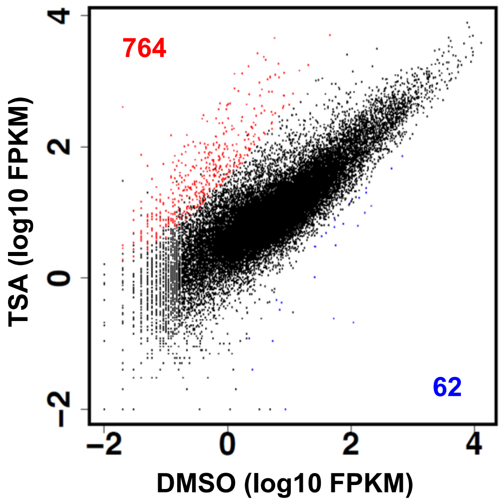

B

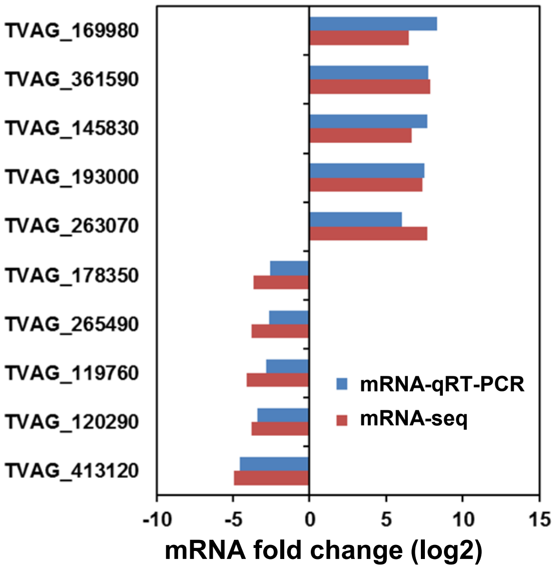

C

Term	Number of genes	P value
oxidation reduction	10	5.9×10^{-5}
regulation of transcription	17	8.7×10^{-4}
cyclic nucleotide biosynthetic process	6	0.01
cyclic nucleotide metabolic process	6	0.01
nucleoside monophosphate biosynthetic process	6	0.01
nucleoside monophosphate metabolic process	6	0.01
nitrogen compound biosynthetic process	8	0.03

D

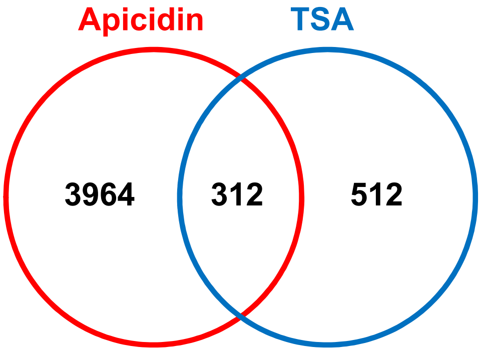

Supplementary Figure 2

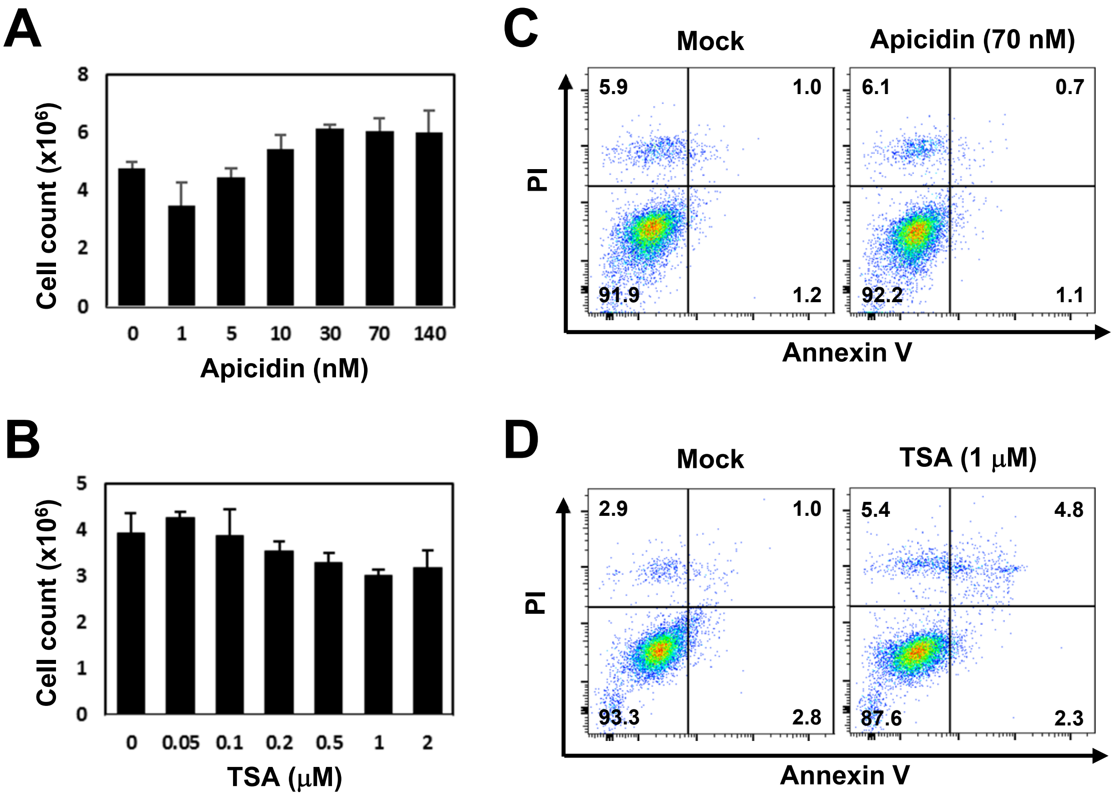

Supplementary Figure 3

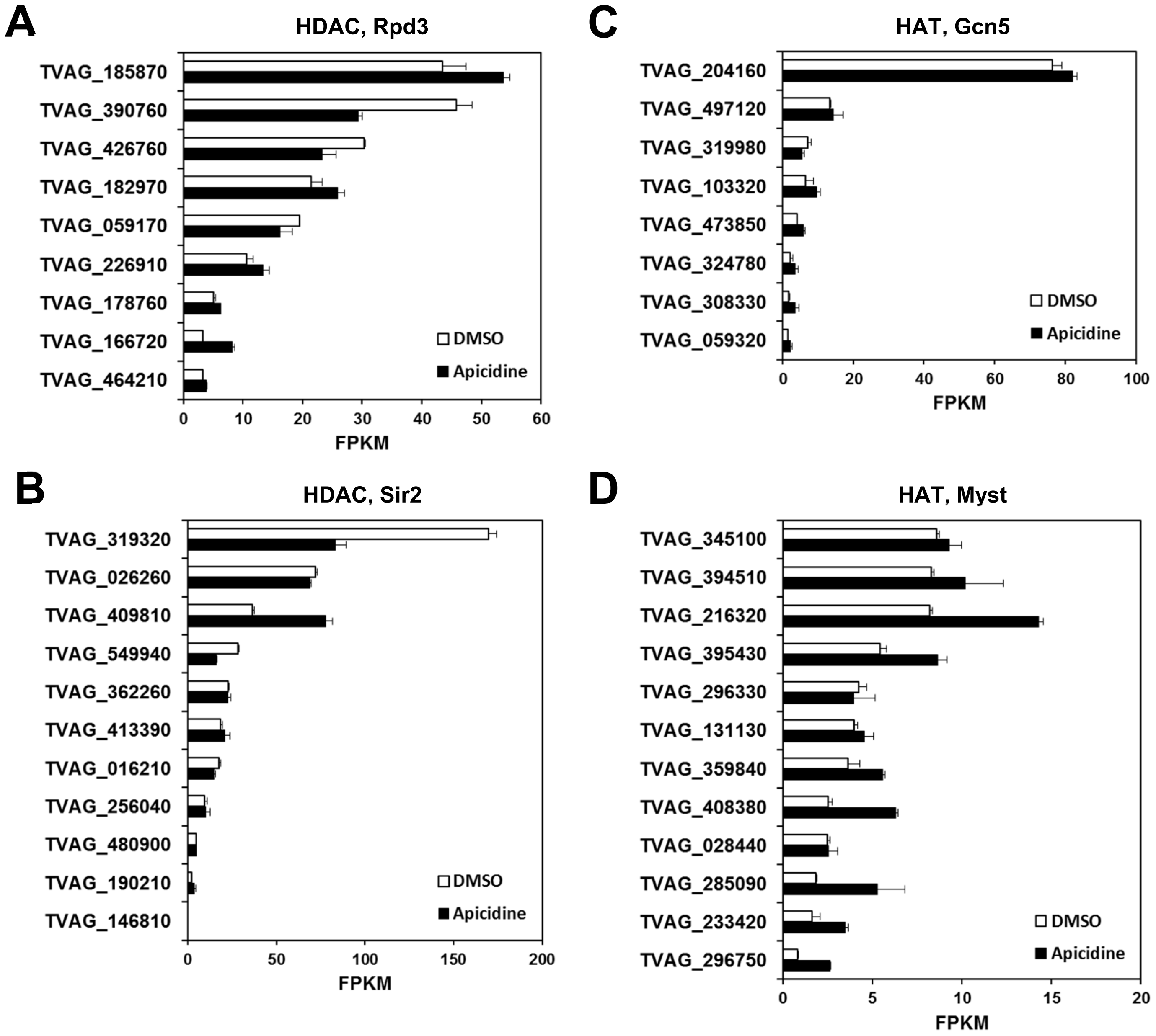

Supplementary Figure 4

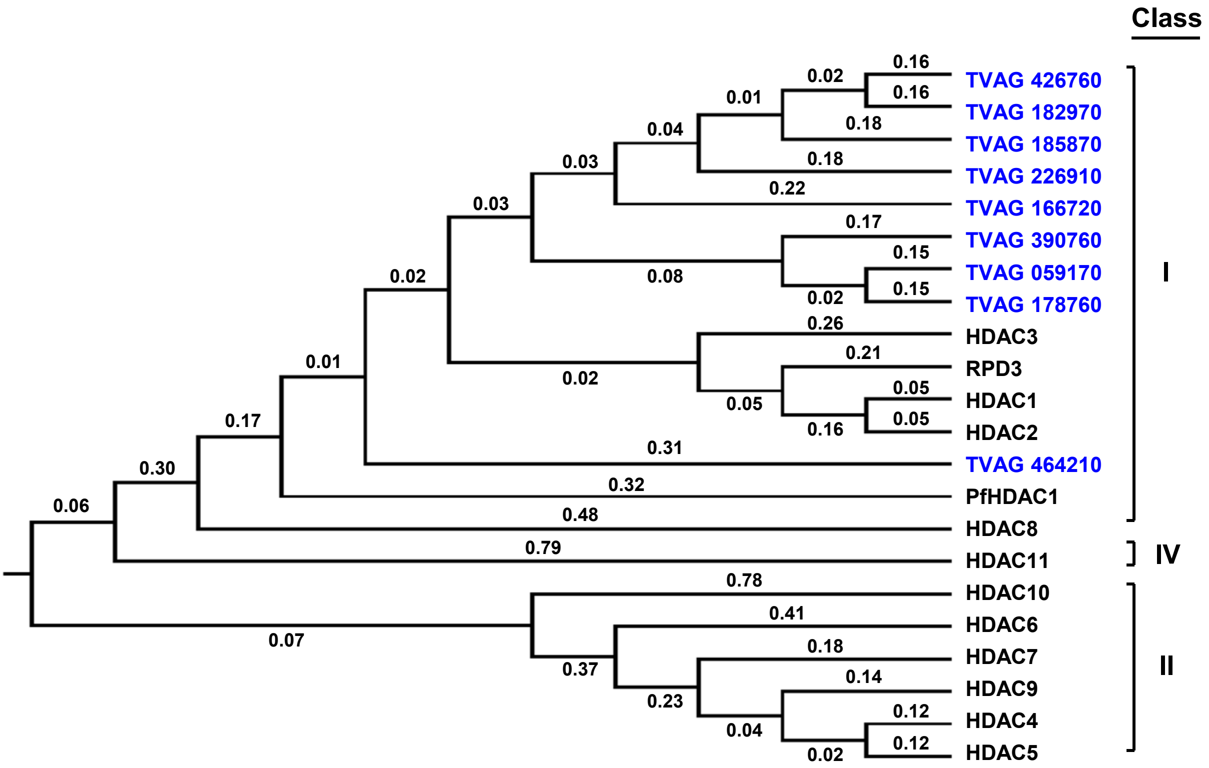

Supplementary Figure 5

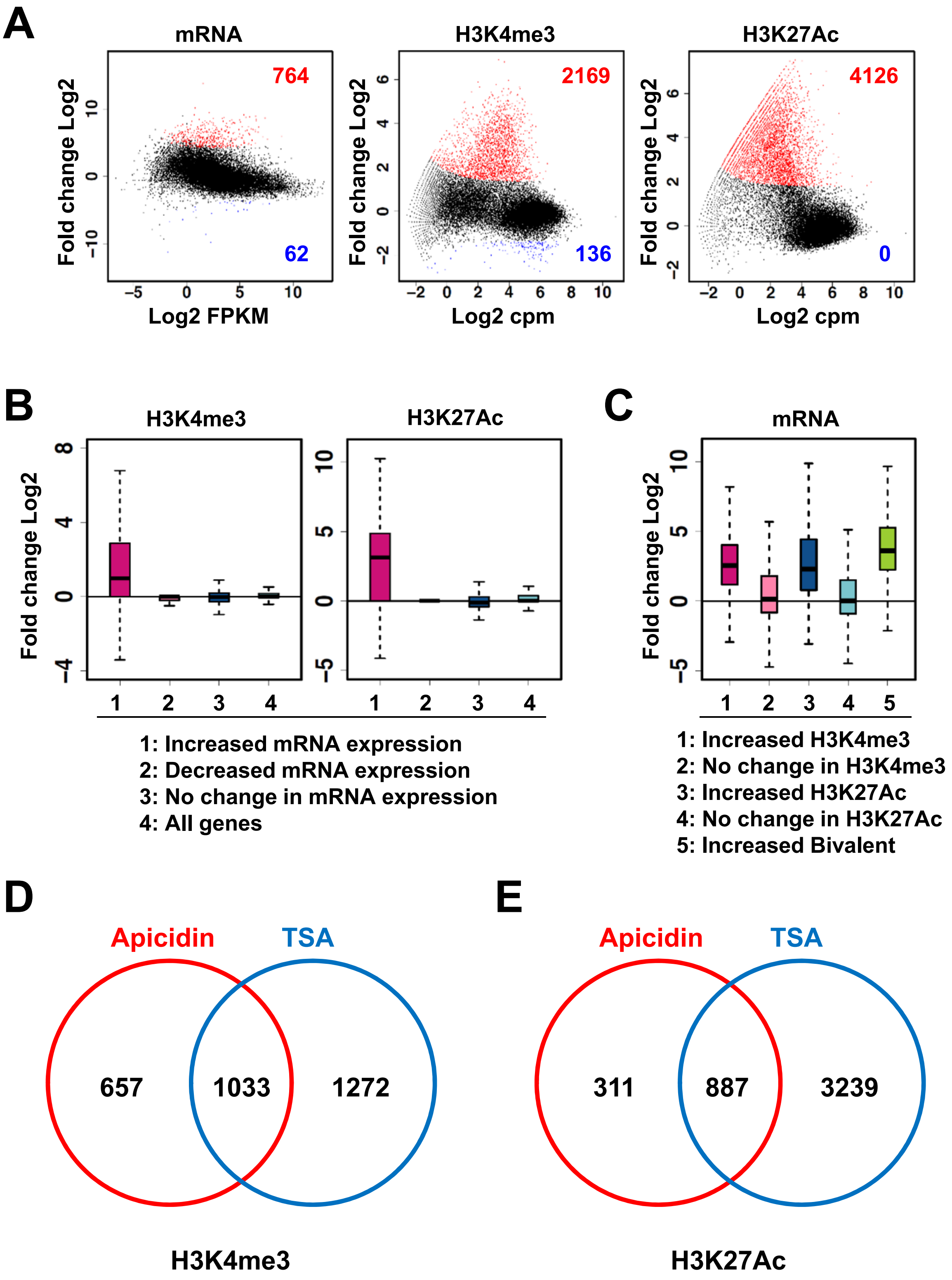

Supplementary Figure 6

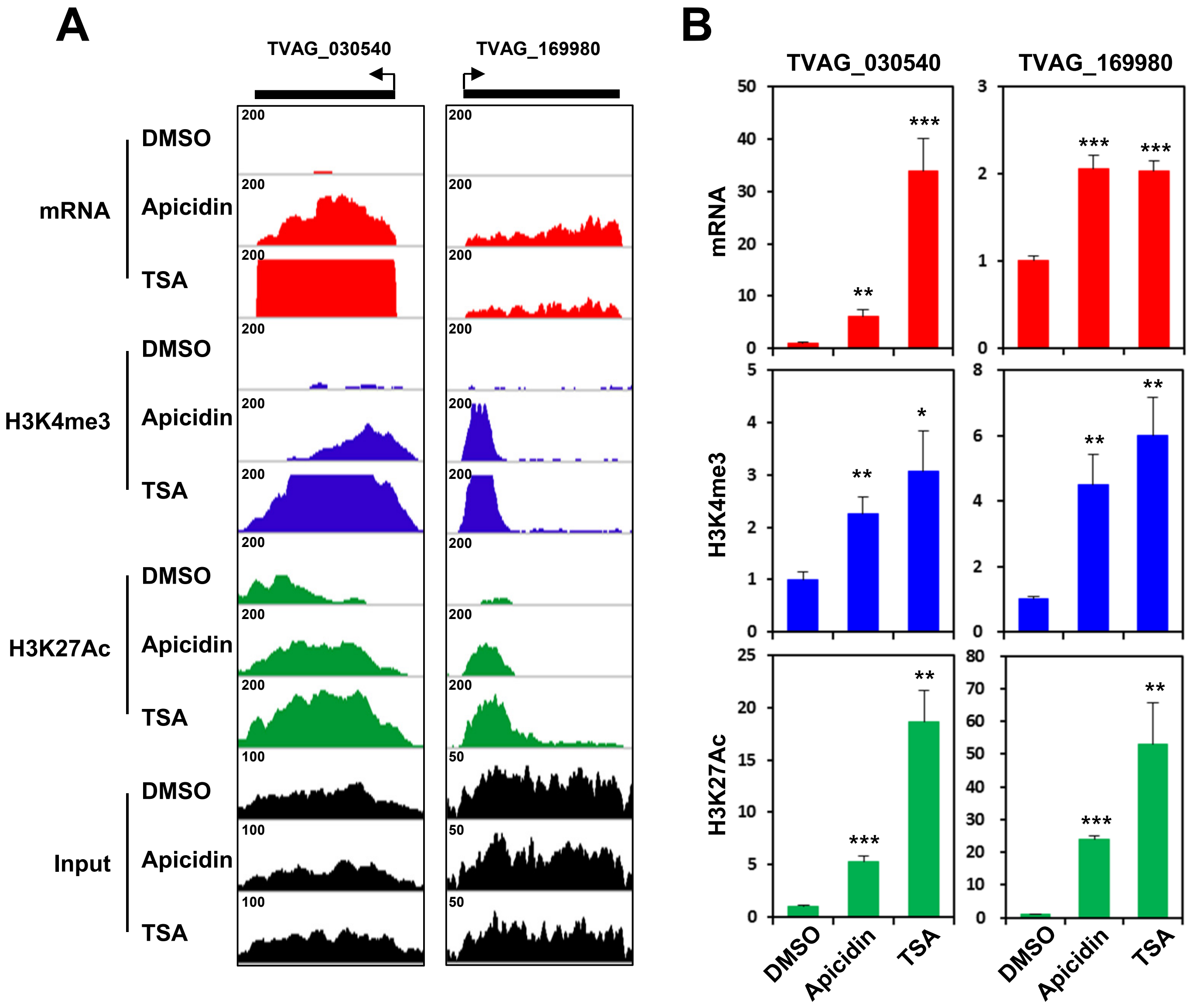

Supplementary Figure 7

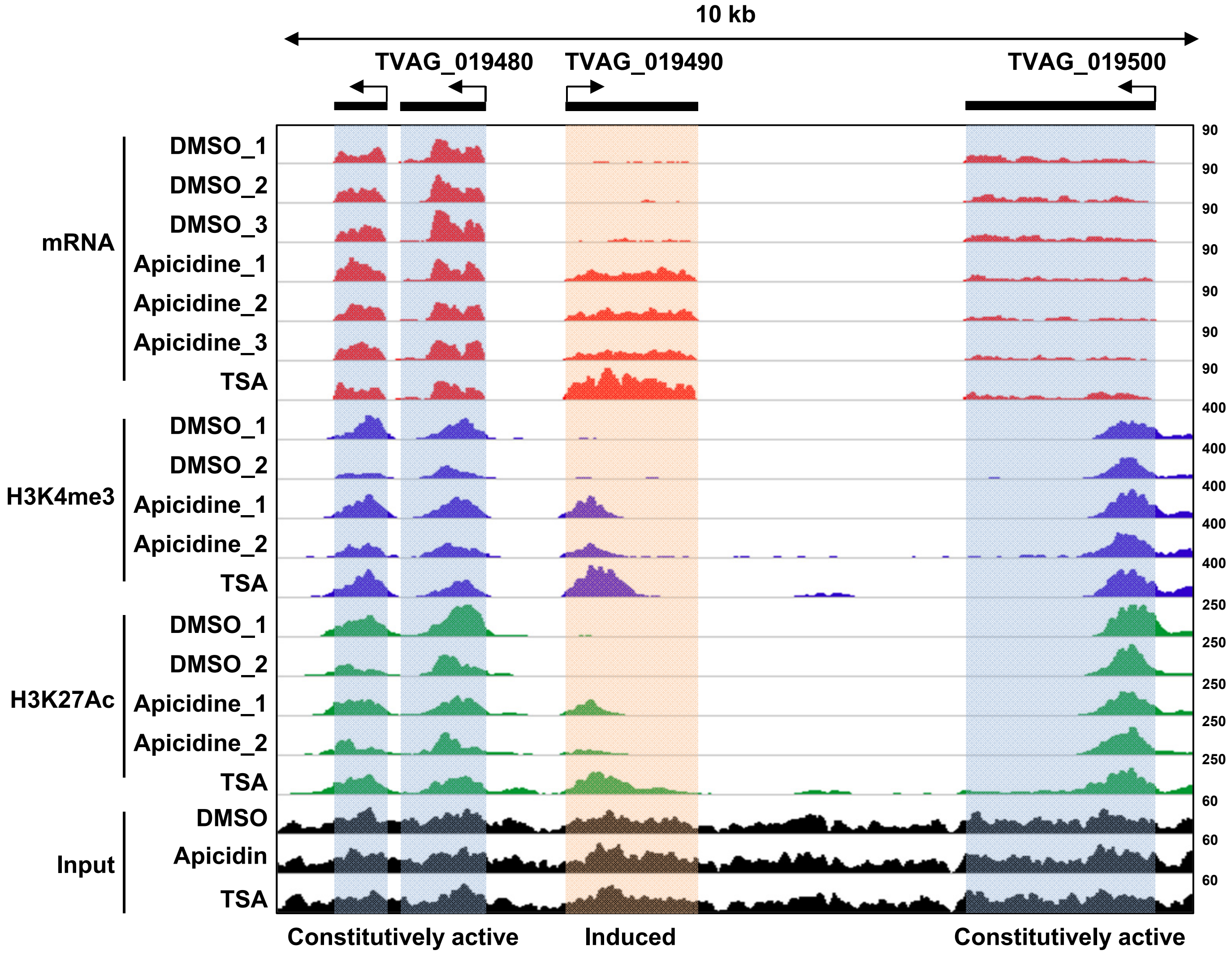

Supplementary Figure 8

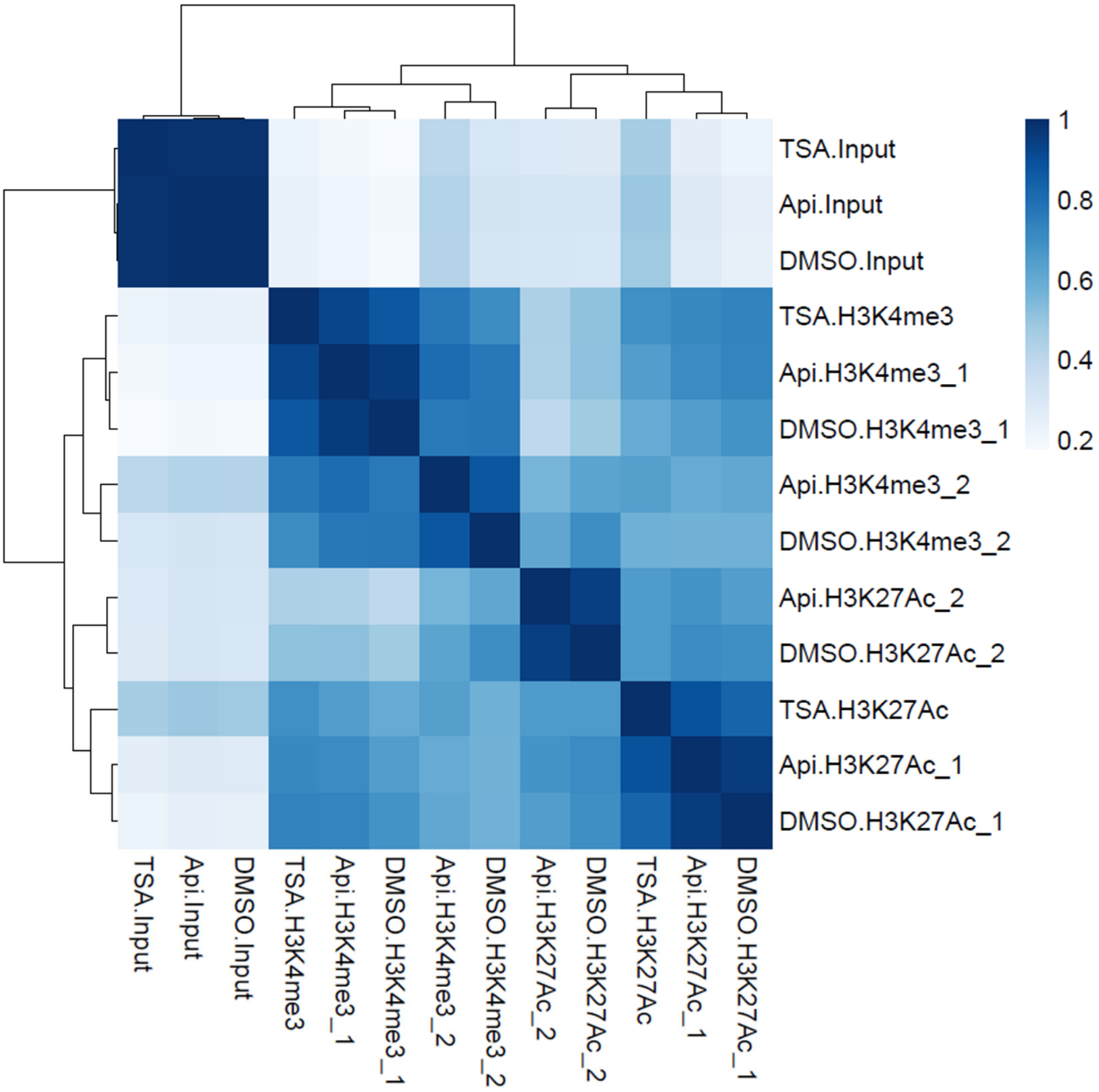

Supplementary Figure 9

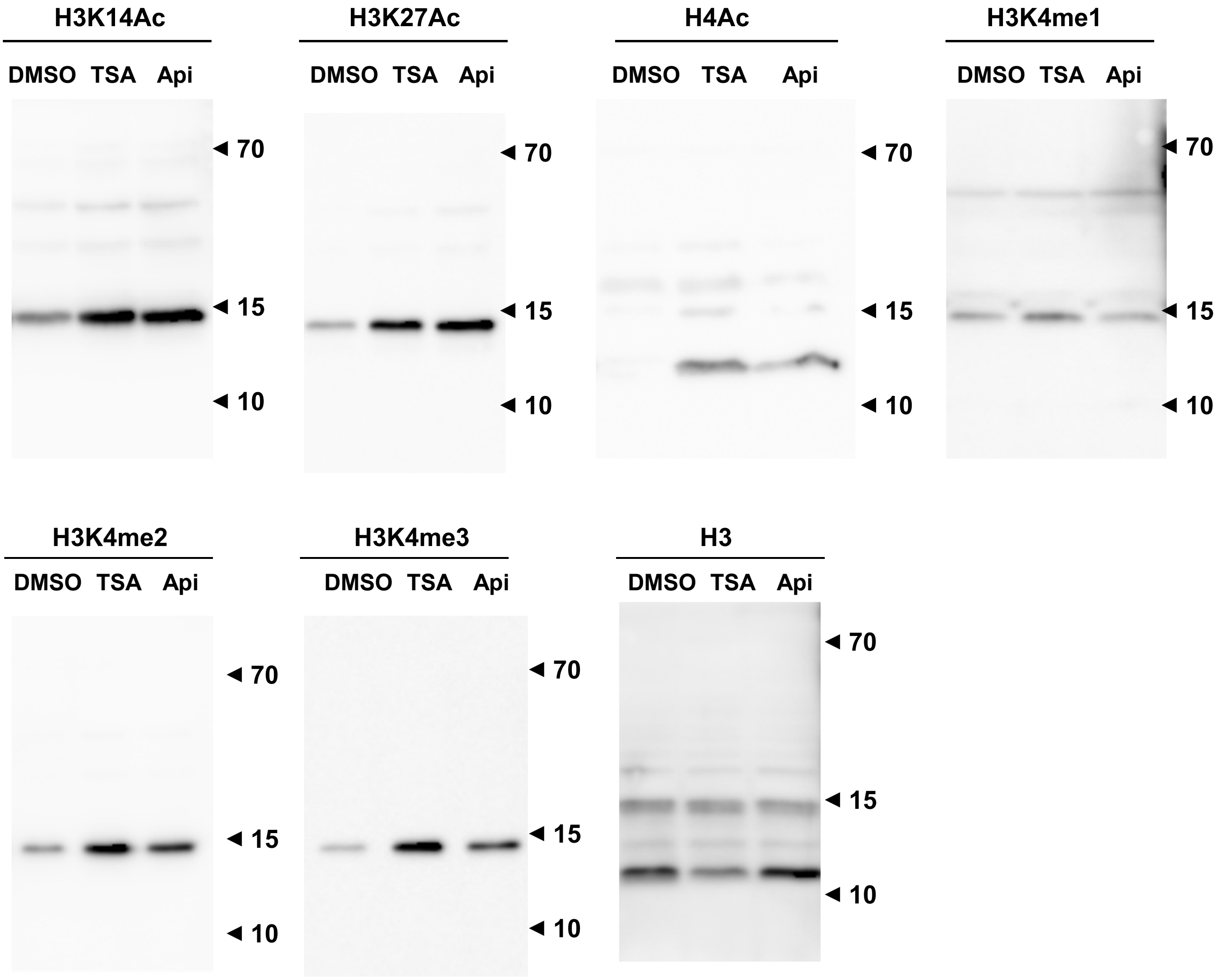

Supplementary Table 1. Putative histone deacetylase enzymes in *T. vaginalis.*

Supplementary Table 2. Putative histone acetyltransferase enzymes in *T. vaginalis.*

Supplementary Table 3. Number of genes categorized by mRNA expression, H3K4me3, or H3K27Ac enrichment in DMSO-treated *T. vaginalis*.

Supplementary Table 4. Primers for qRT-PCR and ChIP-qPCR

Supplementary Table 5. Summary of RNA-seq data from *T. vaginalis*

Supplementary Table 6. Summary of ChIP-Seq data from *T. vaginalis*

Supplementary Table 1. Putative histone deacetylase enzymes in *T. vaginalis.*

Gene identifier	Gene name	HDAC class	No. of amino acids	Molecular weight [kDa]
TVAG_185870	histone deacetylase, putative	I	435	50
TVAG_390760	histone deacetylase, putative	I	453	52
TVAG_426760	histone deacetylase, putative	I	394	44
TVAG_182970	histone deacetylase 1, 2 ,3, putative	I	430	49
TVAG_059170	histone deacetylase, putative	I	418	47
TVAG_226910	histone deacetylase, putative	I	415	46
TVAG_178760	histone deacetylase, putative	I	425	48
TVAG_166720	histone deacetylase 1, 2 ,3, putative	I	417	47
TVAG_464210	histone deacetylase 1, 2 ,3, putative	I	405	46
TVAG_319320	chromatin regulatory protein sir2, putative	III	369	41
TVAG_026260	chromatin regulatory protein sir2, putative	III	375	42
TVAG_409810	chromatin regulatory protein sir2, putative	III	331	37
TVAG_549940	chromatin regulatory protein sir2, putative	III	332	37
TVAG_362260	chromatin regulatory protein sir2, putative	III	312	35
TVAG_413390	chromatin regulatory protein sir2, putative	III	304	34
TVAG_016210	chromatin regulatory protein sir2, putative	III	267	30
TVAG_256040	chromatin regulatory protein sir2, putative	III	281	31
TVAG_480900	chromatin regulatory protein sir2, putative	III	320	37
TVAG_190210	chromatin regulatory protein sir2, putative	III	347	39
TVAG_146810	chromatin regulatory protein sir2, putative	III	180	21

Supplementary Table 2. Putative histone acetyltransferase enzymes in *T. vaginalis.*

Gene identifier	Gene name	HAT subclass	No. of amino acids	Molecular weight [kDa]
TVAG_204160	histone acetyltransferase gcn5, putative	GNAT	156	18
TVAG_497120	histone acetyltransferase gcn5, putative	GNAT	395	46
TVAG_319980	histone acetyltransferase gcn5, putative	GNAT	409	47
TVAG_103320	histone acetyltransferase gcn5, putative	GNAT	188	21
TVAG_473850	histone acetyltransferase gcn5, putative	GNAT	393	46
TVAG_324780	histone acetyltransferase gcn5, putative	GNAT	404	47
TVAG_308330	histone acetyltransferase gcn5, putative	GNAT	359	41
TVAG_059320	histone acetyltransferase gcn5, putative	GNAT	383	45
TVAG_345100	tip60, putative	MYST	376	44
TVAG_394510	myst histone acetyltransferase, putative	MYST	352	41
TVAG_216320	myst histone acetyltransferase, putative	MYST	387	45
TVAG_395430	myst histone acetyltransferase, putative	MYST	380	45
TVAG_296330	myst histone acetyltransferase, putative	MYST	199	24
TVAG_131130	myst histone acetyltransferase, putative	MYST	387	45
TVAG_359840	tip60, putative	MYST	381	44
TVAG_408380	mst1, putative	MYST	373	43
TVAG_028440	myst histone acetyltransferase, putative	MYST	361	43
TVAG_285090	myst histone acetyltransferase, putative	MYST	364	42
TVAG_233420	mst1, putative	MYST	375	44
TVAG_296750	myst histone acetyltransferase, putative	MYST	380	44

Supplementary Table 3. Number of genes categorized by mRNA expression, H3K4me3, or H3K27Ac enrichment in DMSO-treated *T. vaginalis*.

		Number of genes		
Rank	Category	mRNA expression[1]	H3K4me3 enrichment[2]	H3K27Ac enrichment[3]
1	Top 25%	8641	7929	7319
2	25-50%	8642	7928	7308
3	50-75%	8635	7912	7321
4	75-100%	8643	7946	7320
5	None	23297	26081	28528

[1] Category for Figures 5A, 5B, and 5D

[2, 3] Category for Figure 5C

Supplementary Table 4. Primers for qRT-PCR and ChIP-qPCR

Gene	Forward Primers (5' to 3')	Reverse Primers (5' to 3')
TVAG_145830	TGGAAGCACTTGGATGTGAA	GGTCGCAATGTCAAGGATTT
TVAG_263070	GCGTCCTCATCTGCAAAACT	TGGACAATACCCACCAGGAC
TVAG_169980	TACTTTCCCTGCTTCCGTTG	TCTGTAAGTTGCGTGCCTTG
TVAG_361590	GGCTCCGTTACAACAGGAAA	TTTTAAGGCGCTGAACGAGT
TVAG_193000	CAGTCCAGGGCAAGACTCTC	CTTTGGACCAAGCTTTGGAG
TVAG_265490	CGCAATGAAGTGGAGTCTGA	ATAGCAGCAGACAGAGGCAAC
TVAG_178350	GACAGACATATCGGCAGCAA	AAAGTAGGCGAAGCAATGGA
TVAG_119760	GTCCTCGTCAGCTGTCCACT	TGCTCCATGGTCAGAGATTG
TVAG_120290	GATGGAGTTTTGCGTCCTTC	GTCAAGGCCAAGATCCAAGA
TVAG_413120	CAACAGGGGATTGAGGCTAA	CTCCATAGACGAACGCTTGA
TVAG_340290	CCAACGAGCGTGAGAAGAAC	GTAGATGCCGCGGTATGATT
TVAG_198110	ATCAACACATACGAAGAACT	TACTGGTATGGCTTGTAGTA
TVAG_037570	CTACAAGGAAGGTACAGTCA	CAGCTTCTTCACAGATTGTA
TVAG_030540	CAAGGACATTAGGGTTTTCTGG	GATCACACGGATGCATTCAC
TVAG_169980	ATCCGGAAGTAGATACTTTAATTTCA	CCTATGAGAAAACCGCTTGG
TVAG_034440 (β-tubulin)	CAACACAACAGCCTTCCGTG	TGTCATGTTGGAGCGAGCTT

Supplementary Table 5. Summary of RNA-seq data from *T. vaginalis*

Treatment	Total reads	Total high-quality reads	Uniquely mapped reads	Total alignment rate
DMSO_1	35,438,596	33,954,450	20,645,952	60.80%
DMSO_2	33,946,930	32,640,974	19,882,902	60.91%
DMSO_3	40,041,562	38,391,428	23,139,228	60.27%
Apicidin _1	40,601,302	38,781,550	23,662,606	61.02%
Apicidin _2	35,899,078	34,431,102	21,154,268	61.44%
Apicidin _3	34,216,190	32,803,802	20,080,190	61.21%
TSA	39,218,582	37,555,128	23,036,922	61.34%

Supplementary Table 6. Summary of ChIP-Seq data from *T. vaginalis*

Treatment	Marker	Total reads	Total high-quality reads	Uniquely mapped reads	Total alignment rate
DMSO_1	H3K4me3	62,988,391	61,002,414	33,885,955	74.69%
DMSO_2	H3K4me3	57,225,283	52,766,471	17,075,982	32.36%
Apicidin_1	H3K4me3	46,578,339	45,371,087	33,885,955	74.69%
Apicidin_2	H3K4me3	63,142,627	60,399,075	27,868,402	46.14%
TSA	H3K4me3	68,804,899	67,078,333	47,763,458	71.21%
DMSO_1	H3K27Ac	62,988,391	61,002,414	33,885,955	74.69%
DMSO_2	H3K27Ac	63,207,998	59,251,295	23,319,543	39.36%
Apicidin_1	H3K27Ac	46,578,339	45,371,087	33,885,955	74.69%
Apicidin_2	H3K27Ac	59,935,311	57,179,611	24,952,253	43.64%
TSA	H3K27Ac	67,906,351	66,091,465	46,289,380	70.04%
DMSO	Input	47,573,453	46,521,889	17,499,817	37.62%
Apicidin	Input	59,741,237	58,462,448	22,046,267	37.71%
TSA	Input	54,680,381	53,386,193	19,417,644	36.37%

Supplementary Dataset 1. Genes exhibiting more than two-fold changes in expression between DMSO- and apicidin-treated T. vaginalis with an adjusted p-value less than 0.05.

Supplementary Dataset 2. Genes exhibiting more than two-fold changes in expression between DMSO- and TSA-treated T. vaginalis with a p-value less than 0.05.

Supplementary Dataset 3. Genes exhibiting more than 1.5-fold changes in H3K4me3 levels between DMSO- and apicidin-treated T. vaginalis with an adjusted p-value less than 0.05.

Supplementary Dataset 4. Genes exhibiting more than 1.5-fold changes in H3K27Ac levels between DMSO- and apicidin-treated T. vaginalis with an adjusted p-value less than 0.05.

Supplementary Dataset 5. Genes exhibiting more than 1.5-fold changes in H3K4me3 levels between DMSO- and TSA-treated T. vaginalis with an adjusted p-value less than 0.05.

Supplementary Dataset 6. Genes exhibiting more than 1.5-fold changes in H3K27Ac levels between DMSO- and TSA-treated T. vaginalis with an adjusted p-value less than 0.05.
